# Utilization of Noncontrast Magnetic Resonance Lymphangiography for Selection of Effective Surgical Method in Breast Cancer-Related Lymphedema

**DOI:** 10.3390/medicina59091656

**Published:** 2023-09-14

**Authors:** Joseph Kyu-hyung Park, Nakwon Choi, Jaewon Beom, Jae-Young Lim, Yusuhn Kang, Sun-Young Nam, Yujin Myung

**Affiliations:** 1 Department of Plastic and Reconstructive Surgery, Seoul National University Bundang Hospital, Seoul National University College of Medicine, 300 Gumi-dong, Bundang-gu, Seongnam 07061, Republic of Korea; josephpark@snubh.org (J.K.-h.P.);; 2 Department of Rehabilitation Medicine, Seoul National University Bundang Hospital, Seoul National University College of Medicine, Seongnam 07061, Republic of Korea; 3 Department of Radiology, Seoul National University Bundang Hospital, Seoul National University College of Medicine, Seongnam 07061, Republic of Korea

**Keywords:** breast cancer, lymphedema, MRI, lymphovenous anastomosis, lymphangiography, microsurgery

## Abstract

*Background and Objectives:* When considering surgery for patients with breast cancer-related lymphedema (BCRL), it is crucial to determine which surgery will be most effective for the patient and establish the indications for each surgery. Our study retrospectively compared the results of preoperative noncontrast MR lymphangiography (NMRL) performed on the lymphedematous limb of patients before surgery, with the aim of analyzing whether preoperative NMRL can be used as a criterion for determining the type of surgery. *Materials and Methods:* From January 2020 to June 2022, a total of 138 patients with lymphedema underwent surgery at Seoul National University Bundang Hospital. All patients underwent preoperative NMRL imaging and were classified into stages 1–3 based on the MRI severity index using the authors’ previous reference. Three types of surgery, LVA, LVA + liposuction, and LVA + VLNT, were conducted on all patients. The effectiveness of the surgery was evaluated one year postoperatively using the interlimb volume difference before and after surgery, the fluid volume of the edematous limb measured by bioimpedance spectroscopy, and the subjective satisfaction of the patients through the Lymph Q questionnaire. *Results:* In this study, out of a total of 138 patients, 26 (19%) were MRI stage 1, 62 (45%) were stage 2, and 50 (36%) were stage 3. Of the 83 patients who underwent LVA surgery, the greatest decrease in interlimb volume difference was observed in stage 2 patients, and subjective satisfaction was also the most effective in stage 2. In the case of LVA + liposuction patients, a significant volume decrease and a high satisfaction were observed in stage 3 patients. In the case of LVA + VLNT patients, there was no difference in volume decrease according to the stage, but a greater decrease in body fluid volume was observed as the MRI severity index score increased through BIA. *Conclusions***:** In conclusion, this study demonstrates that NMRL imaging is a useful modality for determining the most effective surgical method and predicting the surgical outcome in patients with lymphedema. This highlights the importance of using NMRL in the treatment planning of lymphedema patients.

## 1. Introduction

Breast cancer-related lymphedema (BCRL) is a common complication of breast cancer treatment, particularly surgery and radiation therapy, and can have a significant impact on a patient’s quality of life. BCRL is characterized by the accumulation of lymphatic fluid in the affected limb, which can lead to swelling, discomfort, and impaired function [1,2,3].

With recent developments in microsurgical techniques, new surgical approaches such as super-microsurgical lymphaticovenular anastomosis (LVA) and vascularized lymph node transfer (VLNT) became available to BCRL patients, providing more precise and minimally invasive procedures with promising results [4,5,6,7]. Currently, many surgical treatment options exist for managing BCRL, ranging from physiological operations such as LVA and VLNT to debulking surgeries such as liposuction and the Charles procedure. These options can be performed in combinations as well, such as LVA with VLNT, LVA with liposuction, or VLNT with liposuction [8,9].

Selecting the right operative method and accurate indication for BCRL is crucial for achieving optimal outcomes and minimizing risks for the patient [10,11,12,13]. Unfortunately, determining the most effective treatment for individual patients remains a significant challenge. Current surgical algorithms primarily consider clinical examination findings and patient-reported symptoms, which can be subjective and variable [14,15,16].

Imaging technologies such as high-frequency ultrasonography and indocyanine green lymphangiography have traditionally been employed to guide surgical decisions. However, these modalities have limitations, such as operator-dependency and limited depth of penetration [17,18].

**Figure 1:**
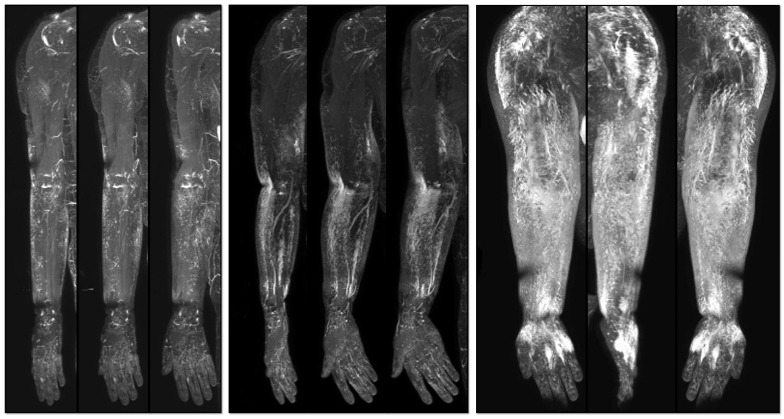
Three-dimensional reconstructed images of noncontrast MR lymphangiography. Left—Stage 1; Middle—Stage 2; Right—Stage 3.

Recent advances in imaging technologies have introduced the use of noncontrast magnetic resonance lymphangiography as a tool for providing detailed imaging of the lymphatic system without the need for contrast agents. In a previous study, we analyzed the lymphedema status of patients using noncontrast magnetic resonance lymphangiography (NMRL) and evaluated the severity and extent of their lymphedema to present the MR staging of lymphedema [19]. Our study found that these stages correlated well with clinical staging, ICG backflow staging, and bioimpedance ratio (Figure 1).

In our clinic, we perform NMRL examinations on all patients before surgery and use them as a reference to determine the appropriate surgical method. To analyze how MRI evaluation can assist in determining the modality of surgery, we retrospectively analyzed the results of patients who underwent various surgical methods for lymphedema based on the stage of their lymphedema diagnosed by MRI before surgery. This analysis allowed us to assess how MRI evaluation can be helpful in determining the most effective surgical approach for each patient.

## 2. Methods

A retrospective cohort analysis was conducted on patients who received management and surgery for BCRL at the Lymphatic Center of Bundang Seoul National University Hospital between January 2020 to June 2022. Patient data, including patient demographics, operative records, limb circumferences, the Lymphedema questionnaire (Lymph Q) [20], and the results of the bioelectrical impedance analysis (BIA) were collected. This study was approved by the Institutional Review Board of Seoul National University Bundang Hospital (B-2105-687-106).

MRI stage and severity index were calculated based on the algorithm previously described [19]. The severity and the extent of involvement at four levels were evaluated: the hand and wrist, the forearm, the elbow, and the upper arm. The calculation of lymphedema severity score was carried out as follows: 0 denoting no abnormalities; 1 indicating dermal thickening accompanied by minimal subcutaneous infiltration; 2 signifying dermal thickening with moderate subcutaneous infiltration and minor perifascial fluid accumulation; 3 representing dermal thickening with severe subcutaneous infiltration and moderate to severe perifascial fluid accumulation. The circumferential extent of the edema was classified as follows: 0, no involvement; 1, involvement of <50% of the circumference; 2, involvement of ≥50% of the circumference. The severity index was the sum of the severity score at all four levels while the extent index was defined as the sum of the extent scores at all four levels. Additionally, a combined index was defined as the sum of the product of the severity score and the extent score at each level.

The primary outcome variables were the preoperative and postoperative 1 year differences in limb volume ratios, BIA, and Lymph Q. The limb volume was calculated based on a formula for a truncated cone and the limb volume ratio was calculated by dividing the volume of the affected limb by the volume of the unaffected limb. The preoperative and postoperative limb volume ratio difference was calculated as the postoperative ratio minus the preoperative ratio. In other words, a positive difference in the limb volume ratio would mean a reduction in the volume of the affected arm. The independent variables were the MRI stage and severity index, as determined by MRL. The relationships were analyzed using analysis of variance (ANOVA). The distributions of outcome variables across different MRI stages and severity indices were visualized using boxplots, stratified by operation type. A *p*-value <0.05 was considered statistically significant, and all statistical analyses were performed using Python 3.7 with SciPy, Pandas, and Pyplot packages.

## 3. Results

A total of 138 BCRL patients were included in this study. The mean age was 51.3 years old, mean body mass index was 24.78 cm/m^2^, and the average history of lymphedema was 4.2 years. Of the 138 patients, 107 previously underwent axillary lymph node dissection while 31 underwent sentinel lymph node biopsy only. Based on our previously defined MRI severity indexing and staging, 26, 62, and 50 patients were categorized as Stage 1, 2, or 3, respectively (Table S1).

**Figure 2:**
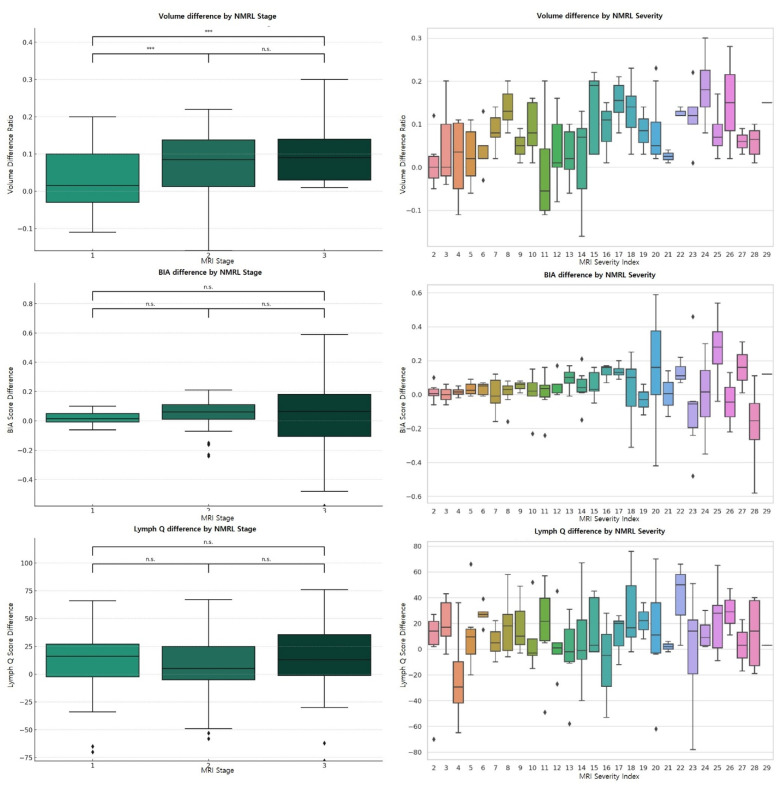
Outcomes by NMRL stage and severity. Volume ratio, BIA score, and Lymph Q score differences are shown for each stage and severity score. (***—*p*-value < 0.05, n.s.—statistically not significant).

Volume ratio improvements were similar between MRI Stages 2 and 3, but greater compared to Stage 1 (Figure 2). However, when plotted against the MRI severity index, there was a positive correlation in terms of limb volume reduction (r = 0.264, *p*-value = 0.00176). On the other hand, BIA score differences among the three stages were similar, while the variability was the greatest in Stage 3. There was no definite trend even when plotted against the MRI severity index. Improvements in the patient-reported outcome (Lymph Q) were also similar between the three MRI stages.

Next, the primary outcome variables were stratified by operation types and MRI stages (Table S2). In Stage 1 (MRI severity index < 7) patients, all underwent LVAs only. In Stage 2 (MRI severity index of 7~17) patients, 40 underwent LVA, 12 underwent LVA and liposuction, while 10 underwent LVA and VLNT. In Stage 3 (MRI severity index of ≥18) patients, 17 underwent LVA only, 15 underwent LVA and liposuction, while 18 underwent LVA and VLNT.

In the LVA-only group, outcomes varied depending on the MRI stage. Stage 3 patients showed the greatest decrease in the volume ratio (−0.083) and the most improvements in Lymph Q scores (14.2). Stage 2 patients showed the greatest decrease in BIA (−0.031). In the LVA and liposuction group, Stage 2 and 3 showed similar improvements in volume ratio and BIA differences. However, Stage 3 patients exhibited higher postoperative satisfactions (Lymph Q difference of 13.5 vs. 11.5). In the LVA and VLNT group, all three outcome variables showed similar improvements between Stages 2 and 3.

**Figure 3:**
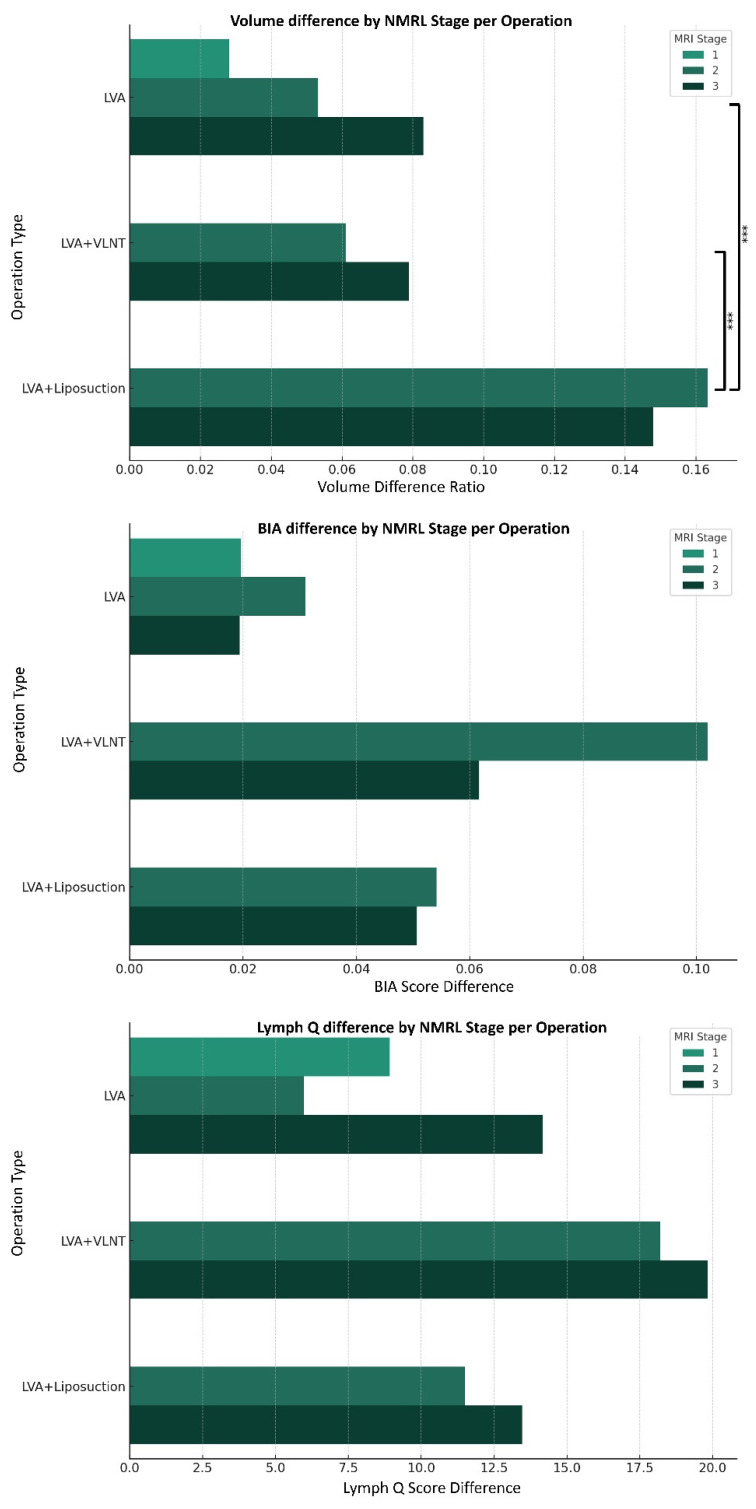
Outcomes for each NMRL stage stratified by operation type. (***—*p*-value < 0.05).

For further analysis on the effectiveness of each surgical method on each MRI stage, outcomes were stratified by operation method (Figure 3). Since all Stage 1 patients underwent LVA only, only Stages 2 and 3 were compared. For patients in MRI Stages 2 and 3, LVA-only and LVA and VLNT showed similar improvements in limb volume. In these patients, LVA and liposuction showed the greatest improvements in limb volume. For the BIA differences, there were no significant differences between the three surgical options. Lastly, Lymph Q differences showed statistically significant differences depending on the operation method. In MRI Stage 2, LVA and VLNT showed the greatest improvement in Lymph Q score compared to LVA-only and LVA with liposuction.

## 4. Discussion

When considering surgery for lymphedema patients, their current condition is crucial. Factors such as the stage of lymphedema, degree of lymphatic clearance, functional lymphatic status, and the condition of the lymph nodes are highly likely to affect the degree of symptom relief after surgery [12,21,22,23,24]. These factors are essential in selecting appropriate surgical options and developing a follow-up treatment plan for symptom relief and prevention of recurrence. Therefore, careful evaluation of the patient, considering these factors, is important when contemplating surgery.

Cutting-edge imaging techniques, such as lymphoscintigraphy, indocyanine green (ICG) lymphography, high-frequency ultrasonography, and magnetic resonance (MR) lymphangiography, are now being implemented in the domain of lymphedema [15,18,25,26,27]. This has facilitated the more precise and sensitive identification of lymphatic vessels and collections of lymphatic fluid. In the past, lymphaticovenular anastomosis (LVA) was mainly conducted on patients in the early stages of lymphedema; however, these improved imaging methods have broadened the criteria for LVA to encompass those with advanced lymphedema as well [23].

Nonetheless, some scholars have speculated that LVA may not be sufficient for treating chronic lymphedema patients, particularly those in the later stages of the International Society of Lymphology (ISL) lymphedema classification [26,27,28,29,30]. Past pathophysiological investigations have demonstrated that chronic inflammation and lymphatic fluid stagnation lead to the breakdown of the lymphatic vessels’ pumping function and programmed cell death in lymphatic endothelial cells [31,32,33]. This, in turn, induces tissue fibrosis and gradual pathological alterations in the lymphatic lumen until the lymphatic vessel becomes hardened and nonfunctional. In such situations, creating a bypass via LVA in the distal lymphatic system, where lymphatic flow is insufficient, may not yield lasting results. For such patients with reduced functional capacity, a more physiological procedure like vascularized lymph node transfer (VLNT) could be advantageous. Nonetheless, VLNT by itself does not yield the immediate volumetric reduction benefits of LVA, so these procedures can be combined to enhance both short- and long-term outcomes. Liposuction, however, can rapidly decrease limb volume and enhance patient adherence to pressure garments and compression bandages, but it does not offer physiological benefits postsurgery [28,34,35].

Our analysis indicated that the operation type could be determined based on the MRI stage or severity index. For patients in MRI Stage 1, LVA-only provided improvements in limb volume, BIA, and Lymph Q scores. In MRI Stages 2 and 3, LVA with liposuction provided the greatest improvement in limb volume. Such a result is expected due to the large volume reduction from liposuction. In terms of BIA and Lymph Q scores, LVA with VLNT provided the most benefit among the three operation types. Since LVA provides volume reducing effects while VLNT provides physiological improvements of lymphangiogenesis, the improvements in Lymph Q are expected.

Our study provides initial evidence supporting the use of NMRL in determining the most effective surgical method for BCRL. By considering the MRI stage and severity index, surgeons can potentially select the most appropriate surgical method, leading to improved patient outcomes. Incorporating NMRL into patient consultation and operation decisions could allow for a more personalized approach to BCRL management, taking into account the individual patient’s lymphatic system’s characteristics. This could help to overcome the limitations of current surgical algorithms, which primarily rely on subjective clinical examination findings and patient-reported symptoms.

Compared to traditional imaging modalities such as high-frequency ultrasonography and indocyanine green lymphangiography, NMRL provides a comprehensive, noninvasive imaging of the lymphatic system. It can provide valuable information about the lymphatic architecture and function, which could supplement or even replace some of these traditional modalities in certain clinical scenarios.

Our study has several strengths, including the use of a relatively large sample of BCRL patients and the comprehensive evaluation of surgical outcomes using multiple measures (limb volume, BIA, and Lymph Q). Furthermore, our study is one of the first to explore the utility of NMRL in guiding the selection of surgical methods for BCRL.

However, our study also has some limitations. As a retrospective study, it is subject to potential biases related to data collection and patient selection. In addition, our analysis did not consider other factors that could influence surgical outcomes, such as the duration of lymphedema and the patient’s overall health status. Also, the lack of postoperative NMRL imaging prohibits us from direct comparison of preoperative and postoperative improvements in the MRI staging and severity index. Future research should address these limitations and validate our findings in prospective, randomized controlled trials.

## 5. Conclusions

In conclusion, this study provides preliminary evidence for the utilization of NRML in determining the most effective surgical method for BCRL. The NMRL parameters, specifically the MRI stage and severity index, could guide the selection of surgical interventions, potentially leading to improved patient outcomes. Further research is needed to validate these findings and to explore the potential of NMRL in improving the management of BCRL.

## Data Availability

The datasets generated during and/or analyzed during the current study are not publicly available due to the protection of patients’ personal medical data but are available from the corresponding author on reasonable request.

## Supplementary Materials

The following supporting information can be downloaded at: https://www.mdpi.com/article/10.3390/medicina59091656/s1, Table S1. Demographics. Table S2. Outcomes Stratified by Operation Types and MRI Stages.
