# Supplementary material for: Utilization of Noncontrast Magnetic Resonance Lymphangiography for Selection of Effective Surgical Method in Breast Cancer-Related Lymphedema
Source: Medicina (Kaunas). 2023 Sep 14;59(9):1656. doi: 10.3390/medicina59091656 (PMC10537151; doi:10.3390/medicina59091656)
Supplement: Supplementary file 1 [file medicina-59-01656-s001.zip › medicina-2554022-supplementary.pdf]

Tables

**Table S1. Demographics**		
		n
**Patient number**		138
**Age (years)**		51.3
**BMI (cm/m^2)**		24.78
**History of lymphedema (years)**		4.2
**Previous lymph node surgery**	ALND	107
	SLND	31
**MRI stage**	1	26
	2	62
	3	50

Table S2. Outcomes Stratified by Operation Types and MRI Stages

Operation Type	n (%)	Preop. Volume Ratio	Postop. Volume Ratio	Volume Ratio Difference	Preop. BIA	Postop. BIA	BIA Difference	Preop. Lymph Q	Postop. Lymph Q	Lymph Q. Difference
LVA	83									
MRI Stage 1	26 (31.3%)	1.075	1.047	-0.028	1.055	1.035	-0.020	50.5	41.6	-8.9
MRI Stage 2	40 (48.2%)	1.182	1.129	-0.053	1.183	1.152	-0.031	52.8	46.8	-6
MRI Stage 3	17 (20.5%)	1.288	1.205	-0.083	1.568	1.549	-0.019	58	43.8	-14.2
LVA + Liposuction	27									
MRI Stage 2	12 (44.4%)	1.263	1.1	-0.163	1.235	1.181	-0.054	47.3	35.8	-11.5
MRI Stage 3	15 (55.6%)	1.353	1.205	-0.148	1.619	1.568	-0.05	47.8	34.3	-13.5
LVA + VLNT	28									
MRI Stage 2	10 (35.7%)	1.19	1.129	-0.061	1.234	1.132	0.102	-57.5	39.3	-18.2
MRI Stage 3	18 (64.2%)	1.277	1.198	-0.079	1.676	1.614	0.062	-65.5	45.7	-19.8

* LVA - lymphaticovenular anastomosis / VLNT - vascularized lymphnode transfer / BIA - Bioelectrical impedance analysis
